# CuO nanorods grown vertically on graphene nanosheets as a battery-type material for high-performance supercapacitor electrodes†

**DOI:** 10.1039/d0ra06758j

**Published:** 2020-10-05

**Authors:** Miaomiao Zhai, Ang Li, Jingbo Hu

**Affiliations:** Department of Chemistry, Beijing Normal University Beijing 100875 P. R. China hujingbo@bnu.edu.cn

## Abstract

This work reports the preparation and characterization of the CuO nanorods grown vertically on graphene nanosheets, denoted as CuO/rGO@NF. Graphene is deposited by electrostatic attraction showing the morphology of folded nanosheets, which improves the electrical conductivity of the electrode, while CuO is modified by filtered cathodic vacuum arc technology and subsequent electrochemical oxidation presenting the morphology of nanorods, which increases the contact area of active sites and shortens the ion and electronic diffusion path. The results show that the CuO/rGO@NF electrode deliver an ultrahigh specific capacity (2.51 C cm^−2^ at 2 mA cm^−2^), remarkable rate performance (64.6%) and improved conductivity. A symmetrical supercapacitor is assembled by two identical electrodes, presenting the maximum energy density of 38.35 W h kg^−1^ at a power density of 187.5 W kg^−1^. Therefore, the CuO/rGO@NF electrode can be used as a prospective electrode for energy storage devices. In addition, the whole electrode preparation process is short in time, safe and environmentally friendly, which provides a new idea for the preparation of other electrode materials.

## Abstract 2

The CuO/rGO@NF electrode is prepared by a simple and time-saving method, which has ultrahigh area capacity and excellent rate performance.

## Graphical abstract

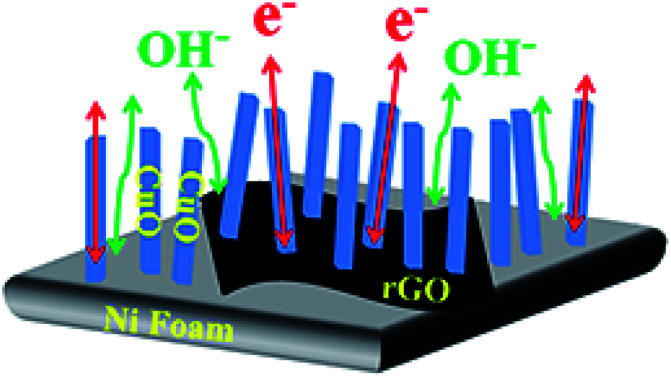

## 1. Introduction

The large demand for portable electronic equipment greatly promotes the development of energy storage devices with small size, flexibility, fast charging and discharging rate, high energy and power density, and long cycle life.^1–3^ Supercapacitors, as a new type of energy storage device that fill the gap of energy density and power density between traditional capacitors and batteries, have attracted great attention in industry and scientific research.^4–6^

The electrode material is an important factor in determining the performance of supercapacitors. The active materials of conventional electrochemical double-layer capacitors (EDLCs) are mainly carbon materials with high specific surface area and excellent conductivity but low capacitance, such as graphene,^7–10^ whose charge storage is realized by electrostatic adsorption. Battery-type electrodes store charges through the Faraday redox process, and the active substances are transition metal compounds, for instance the oxides of copper consisting of CuO,^11–13^ Cu(OH)_2_,^14^ Cu_2_O,^15^ Cu_2+1_O,^16^ which have the advantages of simple preparation, low cost, abundant reserves, many valence states, high electrochemical activity and large capacity, but poor conductivity and stability. At present, the hybrid electrode with transition metal compound and carbon material has gradually become a research hotspot,^17,18^ which could produce synergistic effect between individual materials expecting to have better electrochemical properties. For example, Liu *et al.* fabricated electrostatically charged MoS_2_/graphene oxide hybrid composites with a remarkable cycling stability of 96% capacitance retention after 6800 cycles.^19^ Yuan *et al.* prepared nickel metal–organic framework anchored on graphene oxide (Ni-MOF/rGO) with ultrahigh specific capacitance of 954 F g^−1^ at a current density of 1 A g^−1^.^20^ Therefore, copper oxide and graphene will be introduced into the electrode material in this work. The electrode morphology will be designed reasonably, and the preparation process will be optimized.

As far as the preparation methods of electrode materials of supercapacitors are concerned, traditional hydrothermal method has been widely applied. In order to obtain nanoparticles with superior crystal structure and morphology, high temperature, high pressure, long reaction time, and multiple reaction steps are usually required.^21–23^ Thus, it is difficult to achieve large-scale preparation. For example, Li's group developed hierarchical NiCo_2_O_4_@Ni-MOF hybrid arrays by a two-step synthetic approach including the first step of hydrothermal at 120 °C for 6 h with a post-annealing process at 350 °C for 2 h and the second step of solvothermal process at 120 °C for 12 h.^24^ In order to prepare electrode easily and quickly, filtered cathodic vacuum arc (FCVA) technology are applied, which is a physical substrate surface modification technology using magnetic filtration plasma device. The basic principle of this technique is the use of cathodic vacuum arc discharge to produce a metallic plasma, which selectively enters the vacuum chamber under the control of a magnetic field and then deposits on the surface of the substrate to form a uniform and dense film-modified electrode. Compared with the hydrothermal method, the method has the advantages of fast preparation speed, uniform material distribution, controllability, reproducibility, and optional metal and base materials. For example, our group prepared NiCu_2_S_2_/NF electrodes by filtered cathodic vacuum arc technology and then introduced suitable sulfur by electrochemical process, which deliver outstanding electrochemical activity in energy storage and catalysis.^25^ However, the preparation of hybrid electrodes by this method has not been studied.

Based on the above considerations, the Ni foam electrode modified by graphene and copper oxide (CuO/rGO@NF) was prepared by electrostatic adsorption and filtered cathodic vacuum arc technology with subsequent electrochemical oxidation. The morphology, structure and electrochemical properties of the electrode were characterized. The results showed that the morphology of nanorod-liked copper oxide growing vertically on the graphene nanosheets. The electrode displays ultrahigh area capacity of 2.51 C cm^−2^ at the current density of 2 mA cm^−2^, good rate capability (64.6% capacity retention when current density increased by 7.5 times) and enhanced conductivity. The improved specific capacity and conductivity are the results of synergistic effect of both rGO and CuO due to the complementary. The symmetrical supercapacitor assembled by two CuO/rGO@NF electrodes exhibits high energy density of 38.35 W h kg^−1^ at the power density of 187.5 W kg^−1^. Therefore, this CuO/rGO@NF electrode is superior to similar materials reported in other literatures, and the preparation method is simple and time-saving.

## 2. Experimental section

### 2.1. Materials

Graphene (>98%) was purchased from Beijing InnoChem Science & Technology Co., Ltd. *N*,*N*-Dimethylformamide (HCON(CH_3_)_2_), potassium hydroxide (KOH), ethanol (CH_3_CH_2_OH), hydrochloric acid (HCl), acetone (CH_3_COCH_3_) were gained from Beijing Chemical Reagents Co. Ltd. All the chemicals were of analytical reagent grade and were used as received without further purification. Triple-distilled water was used throughout all experiments. Ni Foam (30 mg cm^−2^, 0.5 mm in thickness) was purchased from Shenzhen Lifeixin Reagent Co. Ltd and was washed ultrasonically with hydrochloric acid (3 mol L^−1^), acetone and distilled water for 20 min each before use.

### 2.2. Fabrication of rGO@NF

Firstly, the Ni foam was soaked in hydrochloric acid solution (3 mol L^−1^) for 2 h to make the surface positively charged. The negatively charged graphene dispersion (4 mg ml^−1^) was obtained by dispersing graphene in *N*,*N*-dimethylformamide (DMF) and sonicating for 2 hours. After that, the Ni foam with positive charges was immersed in the negatively charged graphene dispersion for 0.5 h and then dried in an oven for 2 h. This “immersion and drying” process was repeated three times so that the graphene could be uniformly attached to the substrate to obtain the graphene modified foam nickel electrode (denote as rGO@NF). The mass loading of graphene on Ni foam was about 0.15 mg cm^−2^.

### 2.3. Fabrication of CuO/rGO@NF

The copper film was deposited on the rGO@NF electrode by using the Beijing Normal University (BNU) metal vapour vacuum arc (MEVVA) implanter under the conditions of 90 A, 0 V. In addition, both sides of the electrode were modified for 10 min respectively. In this way, the Ni Foam electrode modified by graphene and Cu was obtained (denote as Cu/rGO@NF)·CuO/rGO@NF was prepared by electrochemical oxidation of copper film. Cyclic voltammetry oxidation was carried out for different time (1, 10, 30, 60 min) at a voltage range of 0–0.6 V and a scan rate of 20 mV s^−1^ in 6 M KOH using Cu/rGO@NF as the working electrode, a platinum wire as the counter electrode, and a Hg/HgO as the reference electrode. Finally, the electrodes were dried at 70 °C in an oven for overnight, and the electrodes oxidized at different time denoted as CuO-01/rGO@NF, CuO-10/rGO@NF, CuO-30/rGO@NF (CuO-30/rGO@NF is abbreviated as CuO/rGO@NF because of its good electrochemical performance), CuO-60/rGO@NF, respectively. The mass loading of copper oxide and graphene on Ni foam was about 0.4 mg cm^−2^.

### 2.4. Characterization

The morphology of the electrode was observed by a field emission scanning electron microscopy (FE-SEM, Hitachi S-8010, Japan) operated at 10 kV and 10 μA. The composition and content of the electrode were detected by energy dispersive X-ray spectrometer (EDS) ran at 15 kV. The crystal phase of the active material on the electrode was tested by X-ray diffraction spectroscopy (XRD, Rigaku Dmax-B, Japan) with a Cu Kα source manipulated at 40 kV and 40 mA. The elements and valence states contained in the electrode were detected by X-ray photoelectron spectroscopy (XPS, Kratos AXIS-Ultra, UK).

### 2.5. Electrochemical measurements

The electrochemical testing of electrodes was carried out on a CHI760E electrochemical workstation in 6 M KOH solution at room temperature using the modified Ni foam electrode (0.5 cm × 1 cm) as the working electrode, a platinum wire as the counter electrode, and the Hg/HgO electrode as the reference electrode. In addition, test methods include cyclic voltammetry (CV), galvanostatic charge–discharge (GCD) and electrochemical impedance spectroscopy (EIS). A symmetric supercapacitor (ASC) device was assembled by using two same CuO/rGO@NF electrodes, and tested in a two-electrode system.

## 3. Results and discussion

**Scheme 1:**
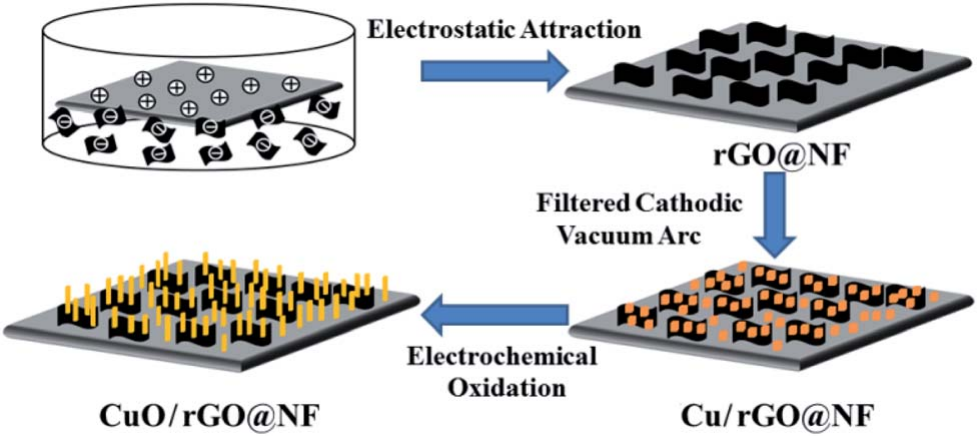
Schematic diagram of preparation of CuO/rGO@NF electrode.

Scheme 1 shows a schematic diagram of the preparation of the CuO/rGO@NF electrode. In advance, The Ni foam was immersed in the hydrochloric acid so that Ni atoms on the surface reacted with H^+^ in to form positively charged Ni^2+^. The graphene was dispersed with DMF to form a negatively charged graphene dispersion solution,^7,19^ and then the positively charged nickel foam was immersed in it to make the graphene nanosheets uniformly adhere to the Ni foam through electrostatic attraction, which increased the surface area and electrical conductivity of Ni foam. Next, copper nanoparticles were injected onto the surface of the graphene using filtered cathodic vacuum arc technology, which was then electrochemical oxidized into copper oxide nanorods.

**Fig. 1:**
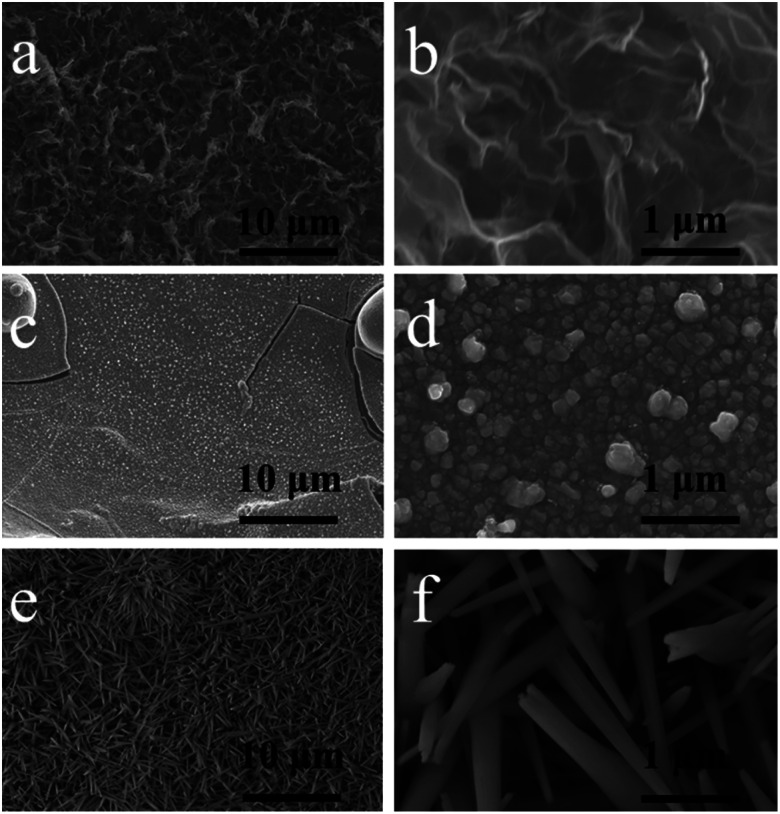
SEM images of (a and b) rGO@NF, (c and d) Cu/rGO@NF and (e and f) CuO/rGO@NF.

The electrode morphology at different stages in the preparation process was observed by SEM. Compared with the bare Ni foam electrode with flat surface morphology (Fig. S1†), the Ni foam surface on the rGO@NF electrode is distributed with abundant pleated graphene nanosheets shown in Fig. 1a and b, which can increase the surface area and conductivity of Ni foam. In Fig. 1c and d, it can be seen that Cu/rGO@NF electrode was prepared by injecting Cu nanoparticles with sizes of 100–200 nm on the graphene layer. Fig. 1c and d shows that Cu nanoparticles after electrochemical oxidation grow into CuO nanospins with 80–280 nm in diameter and extending into any directions, which provide abundant electrochemical active sites and channels for ion diffusion and electron conduction.

In order to gain an insight into the growth mechanism of CuO nanorods, SEM tests were carried out on the electrodes prepared at different electrochemical oxidation times. It can be seen from Fig. S2(a–c)† that after 1 min of electrochemical oxidation (CuO-01/rGO@NF), Cu nanoparticles begin to aggregate and dissolve, and the distinct morphology of nanoparticles disappeared. After 10 min of oxidation, the CuO-10/rGO@NF electrode materials gradually grow in a vertical direction showing the morphology of vertical accumulation of nanoblocks presented in Fig. S2(d–f).† In the process of 30 min oxidation (CuO-30/rGO@NF), the vertical nanoblocks split and form nanorods in Fig. S2(g–i).† When the time extended to 1 h (CuO-60/rGO@NF), nanorods further split resulting in a smaller diameter and a higher density in Fig. S2(j–l).† Therefore, the growth process of the CuO electrode material can be considered as a mechanism of “dissolution and regrowth”.

**Fig. 2:**
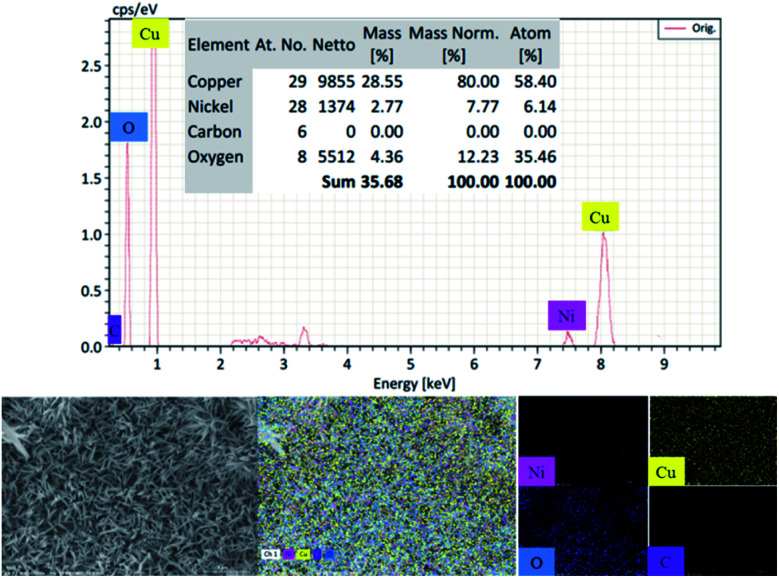
EDS image of the CuO/rGO@NF electrode with different element mapping images, including Ni, Cu, O, C, respectively.

The elements contained in the electrodes and their distributions were characterized by EDS. The results in Fig. 2 show that the CuO/rGO@NF electrode is composed of Cu, Ni, C, O elements, and these elements are evenly distributed on the electrodes. It is worth noting that the content of C is lower than that of other elements, which is due to the masking effect of CuO growing on the surface of graphene, which further proves that the electrode has been successfully prepared. The EDS results for the rGO@NF and Cu/rGO@NF electrodes are presented in Fig. S3.† The rGO@NF electrode consists of Ni and C elements, while the Cu/rGO@NF electrode has a large amount of Cu in addition to Ni and C.

**Fig. 3:**
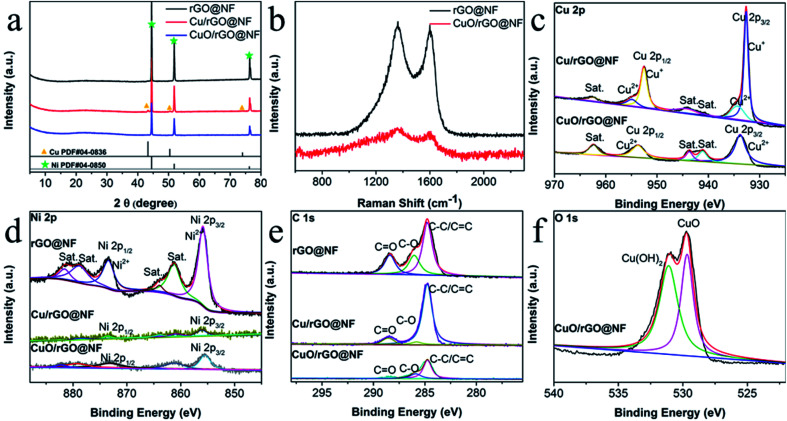
(a) XRD patterns of rGO@NF, CuO/rGO@NF and CuO/rGO@NF electrodes, (b) Raman spectrum of rGO@NF and CuO/rGO@NF, (c) the Cu 2p, (d) the Ni 2p, (e) the C 1s and (f) the O 1s XPS spectra of all electrode.

The crystal phase of the electrode material was characterized by XRD, and the results were shown in Fig. 3a. The three major peaks at 44.5°, 51.8° and 76.4° are attributed to the (111) (200) and (220) planes of Ni foam (JCPDS no. 04-0850). The other three diffraction peaks 43.3°, 50.4° and 74.1° correspond to (111) (200) and (220) planes of Cu elemental (JCPDS no. 04-0836). However, the diffraction peak of CuO phase is not obvious, which may be because the amount of CuO relative to Ni foam substrate is very small, resulting in the diffraction peak almost level with the baseline. In addition, there is a broad peak at 23°, which is indexed to the (002) plane of graphene.^26^

Raman spectrum presented in Fig. 3b shows two typical D-band and G-band peaks at 1357 cm^−1^ and 1593 cm^−1^, respectively. The D-band peak is caused by structural defects and disorder of carbon material, while the G-band peak indicates the degree of carbon graphitization.^19^ The *I*_D_/*I*_G_ value of CuO/rGO@NF is 1.12, which is higher than that of rGO@NF (1.02), indicating that defects of the electrode increase after the introduction of Cu,^27^ which is conducive to improving the conductivity of the electrode.^26^

The chemical composition and elemental chemical valence of the CuO/rGO@NF electrode were further characterized by XPS test. The survey spectrum in Fig. S4† proves that the CuO/rGO@NF electrode contains Ni Cu C O element, which is consistent with the EDX results. The Cu 2p spectrum of CuO/rGO@NF electrode is deconvoluted into Cu 2p_3/2_ at 933.8 eV and Cu 2p_1/2_ at 953.7 eV, which proves that Cu existed as Cu^2+^. In addition, three satellite peaks at 941.1, 943.7 and 962.4 eV are found.^11,28^ The Cu 2p spectrum of Cu/rGO@NF electrode includes the Cu^+^ peak at 932.7 eV and 952.6 eV and the Cu^2+^ peak at 934.27 and 954.9 eV,^25,29^ among which Cu^+^ is the main form. Compared with that before oxidation, the 2p peak of Cu^2+^ in CuO/rGO@NF electrode shifted 1.2 eV to a lower angle, indicating a strong interaction between CuO and rGO after electrode oxidation.^30,31^ In the Ni 2p spectrum, the two main peaks at 855.7 for Ni 2p_1/2_ and 873.5 eV for Ni 2p_3/2_ peaks are assigned to Ni^3+^,^18^ which is derived from the oxidation of Ni foam. The C 1s spectrum is composed of C–C/C=C, C–O, and C=O at 284.7, 286.0, and 288.6 eV respectively,^5,27,31^ demonstrating the presence of graphene in the electrode material. In the 1s peak of O, the two peaks of 529.7 and 531.2 eV respectively represent the existence of CuO and Cu(OH)_2_.^11^

**Fig. 4:**
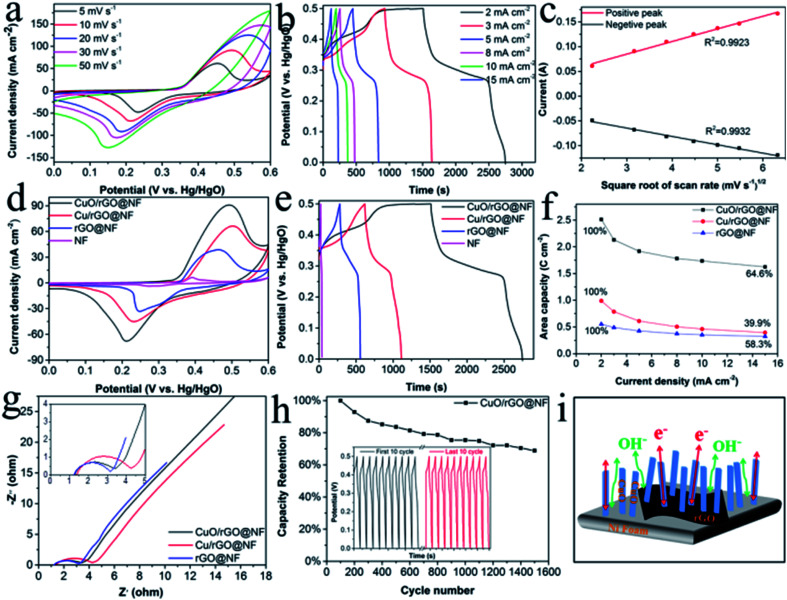
(a) CV curves of the CuO/rGO@NF electrode at different scan rates. (b) GCD curves of the CuO/rGO@NF electrode at various current densities. (c) The linear relationship of the CuO/rGO@NF electrode between the square root of scan rate and the peak current. (d) Comparative CV curves of the NF, CuO/rGO@NF, Cu/rGO@NF and rGO@NF at 10 mV s^−1^. (e) Comparative GCD curves of the four electrodes at 2 mA cm^−2^. (f) Specific capacitance of the CuO/rGO@NF, Cu/rGO@NF and rGO@NF electrodes at different current densities. (g) EIS plots of the three electrodes at a frequency range of 100 kHz–0.01 Hz. (h) Cycling stability of the three electrodes at a current density of 25 mA cm^−2^. (i) Schematic diagram illustrating the merits of the CuO/rGO@NF electrode.

The electrochemical performances of the CuO/rGO@NF (abbreviated as CuO-30/rGO@NF) and other electrodes (NF, rGO@NF and Cu/rGO@NF) were tested with the three-electrode system in the 6 M KOH solution, and the results are shown in Fig. 4a–c and S5.† All CV curves of CuO/rGO@NF electrodes at various scan rates in Fig. 4a show a pair of distinct redox peaks, which are related to faradaic redox reaction as following equation:^11,16,32,33^CuO + H_2_O + 2e^−^ = Cu_2_O + 2OH^−^Cu_2_O + H_2_O + OH^−^ = Cu(OH)_2_ + 2e^−^CuOH + OH^−^ = Cu(OH)_2_ + 2e^−^CuOH + OH^−^ = CuO + H_2_O + e^−^

With the increase of scan rate, the area of CV curve enlarges with remaining the shape unchanged, indicating the ascendant electrochemical reversibility of electrode materials. The GCD curves of CuO/rGO@NF electrodes at different current density in Fig. 4b have obvious platforms, testify the battery-type characteristics of energy storage. The discharge time decreases as the increase of current density owing to the insufficient contact of electrode material with the electrolyte at a higher current density. The area capacity of the CuO/rGO@NF electrode is 2.51, 2.13, 1.92, 1.78, 1.74, 1.62 C cm^−2^ on the basis of eqn (S1)† at the current density of 2, 3, 5, 8, 10, 15 mA cm^−2^, which is comparable with similar electrodes prepared by different methods reported in the previous literature in the Table S1.† The linear relationship of the CuO/rGO@NF electrode between the square root of scan rate and the peak current in Fig. 4c proves that the reaction is diffusion-controlled battery-type behavior,^21,24,34^ indicating that the CuO materials capable of redox reactions plays an important role in charge storage.^35,36^

Electrochemical characterization was performed on the electrode prepared at different oxidation times, and the results in Fig. S6† showed that when the oxidation time was 30 min, the CV curve area of the electrode is the largest, and the discharge time of the GCD curve is the longest. According to the GCD test, the maximum capacities calculated by eqn (S1)† of the electrodes prepared for 1, 10, 30, 60 min are 0.66, 1.31, 2.51, 1.23 C cm^−2^ at the current density of 2 mA cm^−2^. When the current density increased to 15 mA cm^−2^, the capacities retention rate are 39.8%, 57.5%, 64.6% and 40.9%, respectively. It can be seen that the capacities of the electrodes increases first and then decreases with the extension of the oxidation time, which is related to the morphologies change of the electrodes during the oxidation process. During the oxidation process, Cu nanoparticles gradually grow into CuO nanorods vertically arranged on graphene nanosheets. This 3D structure not only increases the contact area between the active material and the electrolyte, but also provides the ion diffusion channel resulting in improved electrical conductivity. However, with excessive oxidation, the copper oxide nanorods grow too densely leading to the narrow interior space, which prevents the ions from diffusing into the electrode material resulting in a decrease in capacity. Therefore, 30 min is the optimal oxidation time, and the prepared electrode is abbreviated as CuO/rGO@NF.

As can be seen from the comparative CV curves (Fig. 4d) of NF, CuO/rGO@NF, Cu/rGO@NF and rGO@NF electrodes at a scan rate of 10 mV s^−1^ with a potential window of 0–0.6 V, the CuO/rGO@NF electrode has the maximum CV curve area, indicating its maximum capacitance. It is worth noting that the CV curve of rGO@NF electrode has redox peaks rather than a rectangular shape as in the previous literature.^7,9^ This may be due to the structural defects in carbon materials and the small load of grapheme,^10^ which increases the conductivity and activation of Ni foam. Thus, the rGO@NF electrode reflects the characteristics of battery-type rather than the electric double-layer capacitors. Fig. 4e shows the GCD curves of NF, CuO/rGO@NF, Cu/rGO@NF and rGO@NF electrodes at a current density of 2 mA cm^−2^ with a potential window of 0–0.5 V. The maximum discharge time of the CuO/rGO@NF electrode proves its excellent capacity derived from its unique 3D structure, and the synergistic effect between the individual materials, while the maximum capacity of NF, Cu/rGO@NF and rGO@NF electrodes is 0.05, 0.55 and 0.99 C cm^−2^, respectively. The capacity retention rate of CuO/rGO@NF electrode is 64.6%, when the current density increases from 2 to 15 mA cm^−2^, which is superior to the Cu/rGO@NF (39.9%) and rGO@NF (58.3%) electrodes, as shown in Fig. 4f.

Electrochemical impedance spectroscopy (EIS) test in the frequency range of 0.01 Hz–100 kHz was conducted to examine the resistance behavior of the CuO/rGO@NF, Cu/rGO@NF, rGO@NF electrode, and the results are presented in Fig. 4g. Internal resistance (*R*_s_) of the electrodes is the intercept on *X*-axis, and the *R*_s_ values of rGO@NF electrode (1.24 Ω) is smaller than that of CuO/rGO@NF (1.29 Ω) and Cu/rGO@NF (1.39 Ω) electrodes. The semicircle in the high frequency region of represents the charge transfer resistance (*R*_ct_), and the *R*_ct_ value of rGO@NF electrode (1.91 Ω) is also smallest resulted from the small resistance of graphene itself. Noteworthily, the *R*_s_ and *R*_ct_ values of CuO/rGO@NF (2.09 Ω) is smaller than Cu/rGO@NF (2.84 Ω) electrodes, testifying CuO nanorods have better conductivity because of the larger area to contact the electrolyte compared with copper nanoparticles. The straight line in the low-frequency region is related to the Warburg impedance (*W*_R_), and the steep inclinations of the three lines suggest rapid ion diffusion, which is favorable for the improvement of capacity.

The cycle life is also a vital factor in determining electrode performance. The stability of the CuO/rGO@NF electrode was tested by performing the GCD test for 1500 cycles at a high current density of 25 mA cm^−2^. The result in Fig. 4h is that the specific capacity of the CuO/rGO@NF electrode remains 68.9% of the original value. The SEM image of CuO/rGO@NF after cycling (Fig. S7b†) shows CuO nanorods and part of graphene are peeled off from the electrode. The separation of the active material from the electrode during long-term test can cause the poor stability, which is also verified by the significantly reduced resistance of the electrode after cycle by EIS test, presented in Fig. S7a.†

In conclusion, the CuO/rGO@NF electrode has outstanding performance, and can be used as a candidate for supercapacitors. This extraordinary performance is due to the following factors: (1) graphene nanosheets adhere to Ni foam by electrostatic attraction, which avoids agglomeration of the nanosheets, and increases the surface area of the electrode. (2) Copper nanoparticles introduced by filtered cathodic vacuum arc technology provide seed for subsequent *in situ* oxidation. The electrochemically oxidized CuO exhibits a nanorod-like morphology arranged vertically on the graphene nanosheets. This unique structure increases the active site contact area, and facilitates the diffusion of ions and electrons into the material, as exhibited in Fig. 4i. (3)There are no binders and conductive agents are involved in the preparation, which reduce the dead volume and improve the conductivity of the electrode. (4) The synergistic effect existing between the intimate contact of CuO, rGO and Ni foam leads to the ultrahigh capacity.

**Fig. 5:**
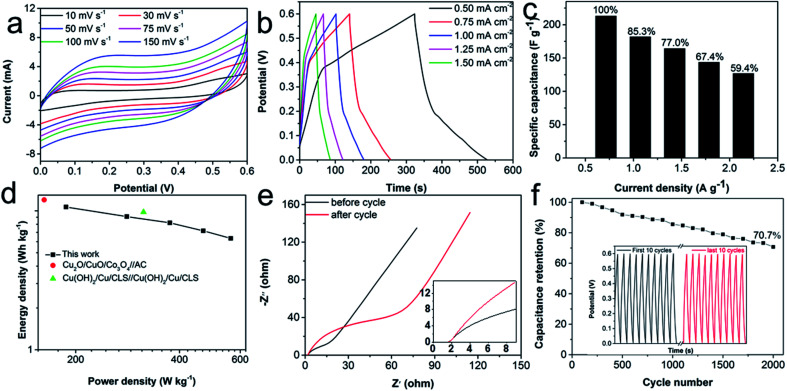
(a) CV curves of the CuO/rGO@NF//CuO/rGO@NF symmetric supercapacitor device at different scan rates. (b) GCD curves of the device at various current densities. (c) Specific capacitance of the device at different current densities. (d) Ragone plot of the device. (e) EIS plots of the device at a frequency range of 100 kHz–0.01 Hz. (f) Cycling stability of the device at a current density of 3 mA cm^−2^.

To validate the practical application of the electrode, a symmetric supercapacitor was assembled by using two identical CuO/rGO@NF electrodes. The electrochemical test results of the device are shown in Fig. 5. As can be seen from Fig. 5a, the area of CV curve increases without changing CV shapes, as the increase of scan rates. GCD curves (Fig. 5b) at different current densities are almost symmetrical triangular curves, proving their rapid charging and discharging behaviors. The specific capacitance of the device is calculated as 213.05 F g^−1^ at the current density of 0.5 mA cm^−2^. When the current density increases to 1.5 mA cm^−2^, the device reserves 59.4% of the initial specific capacitance (Fig. 5c), indicating that the device has a good rate capability. The energy density and power density of the device are also calculated, and the corresponding Ragone plot is shown in Fig. 5d. The maximum energy density of the device reaches 38.35 W h kg^−1^ at power density of 187.5 W kg^−1^ and still maintains at 22.8 W h kg^−1^ even at a high power density of 563.0 W kg^−1^, which is superior to other reported copper and graphene-based literature, *e.g.*, Cu_2_O/CuO/Co_3_O_4_//AC,^37^ Cu(OH)_2_/Cu//Cu(OH)_2_/Cu.^14^ According to the EIS results (Fig. 5f), the *R*_s_ value of the device is small (1.57 Ω), and the linear inclination of the low-frequency area is high, suggesting the ascendant conductivity of the device. In addition, the device proves the superior stability by retaining a specific capacitance of 70.7% after 2000 cycles at a current density of 3 mA cm^−2^ and remaining the same shape of the first 10 and last 10 cycles of the GCD curve (Fig. 5e). The EIS results of the device after the stability test shows that the *R*_ct_ value of the device increased significantly, which is caused by the shedding of active materials during the cycle, which is also the reason why the capacitance of the device decreases after the cycle test.

## 4. Conclusions

In this work, we prepared a Ni foam electrode modified by graphene and copper oxide by electrostatic interaction and filtered cathodic vacuum arc technology with subsequent electrochemical oxidation, denote as CuO/rGO@NF. This electrode exhibits the morphology of nanorod-liked copper oxide vertically grown on graphene nanosheets. Benefiting from the distinctive three-dimensional structure, the improved conductivity, the larger contact area of the electrolyte and the synergistic effect between the electrode materials, the electrode presents a high specific capacity. The symmetrical supercapacitors also demonstrate splendid energy and power densities. Therefore, this electrode is promising for energy storage devices. In addition, the preparation method is time-saving, safe and environmentally friendly, which provides a new idea for the preparation of other electrode materials.

## Conflicts of interest

There are no conflicts to declare.

## Supplementary Material

RA-010-D0RA06758J-s001
