# Supplementary material for: CuO nanorods grown vertically on graphene nanosheets as a battery-type material for high-performance supercapacitor electrodes
Source: RSC Adv. 2020 Oct 5;10(60):36554–61. doi: 10.1039/d0ra06758j (PMC9057026; doi:10.1039/d0ra06758j)
Supplement: RA-010-D0RA06758J-s001 [file RA-010-D0RA06758J-s001.pdf]

Electronic Supplementary Material (ESI) for RSC Advances.
This journal is © The Royal Society of Chemistry 2020

Supporting Information

CuO Nanorods Grown Vertically on Graphene Nanosheets as a Battery-type Material for High-Performance Supercapacitor Electrodes

Miaomiao Zhai[a], Ang Li[a], Jingbo Hu*[a]

[a]College of Chemistry, Beijing Normal University, Beijing 100875, PR China.

*Corresponding author.

E-mail addresses: hujingbo@bnu.edu.cn

Contents of the Supporting Information

Total number of pages: 11

Total number of figures: 7

Total number of tables: 1

The specific capacitance C_A (C cm^{-2}) was calculated from the GCD curves according to the following formula:

$$C_A = I \times \Delta t/(A) \qquad \text{(S1)}$$

where I, Δt, and A corresponds to discharge current (A), discharge time (s), and the area of the electrode (cm^{-2}), respectively.

An symmetric supercapacitor (SSC) device was assembled by using two same CuO/rGO@NF electrodes, which was analyzed under a 6.0 M KOH solution at room temperature. The specific capacitance C_{cell} (F g^{-1}) was calculated from the GCD curves according to the following formula:

$$C_{cell} = I \times \Delta t/(\Delta V \times M) \qquad \text{(S2)}$$

where I, Δt, $\Delta V$and M corresponds to discharge current (A), discharge time (s), potential window (V) and the total mass of active materials of both electrodes (g), respectively. The energy density E (W h kg^{-1}) and power density P (KW kg^{-1}) was performed according to the following equations:

$$E = C_{cell} \times \Delta V^2/2 \qquad \text{(S3)}$$

$$P = E/\Delta t \qquad \text{(S4)}$$

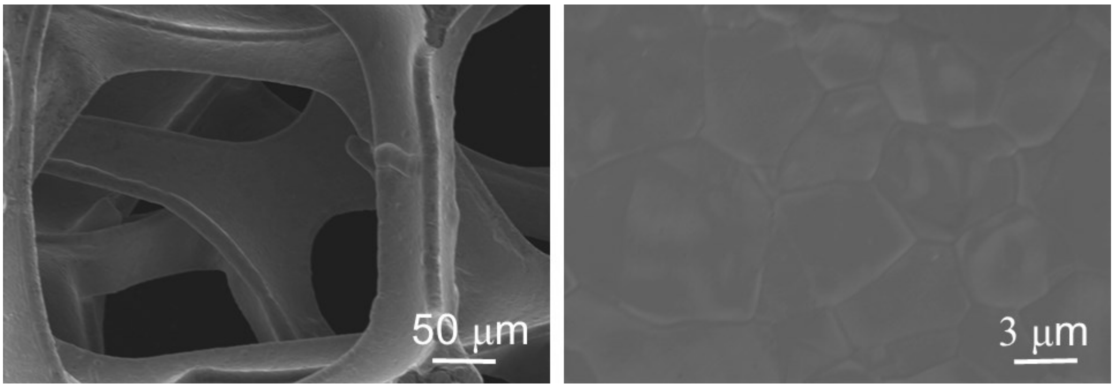

Figure S1. SEM image of bare Ni Foam

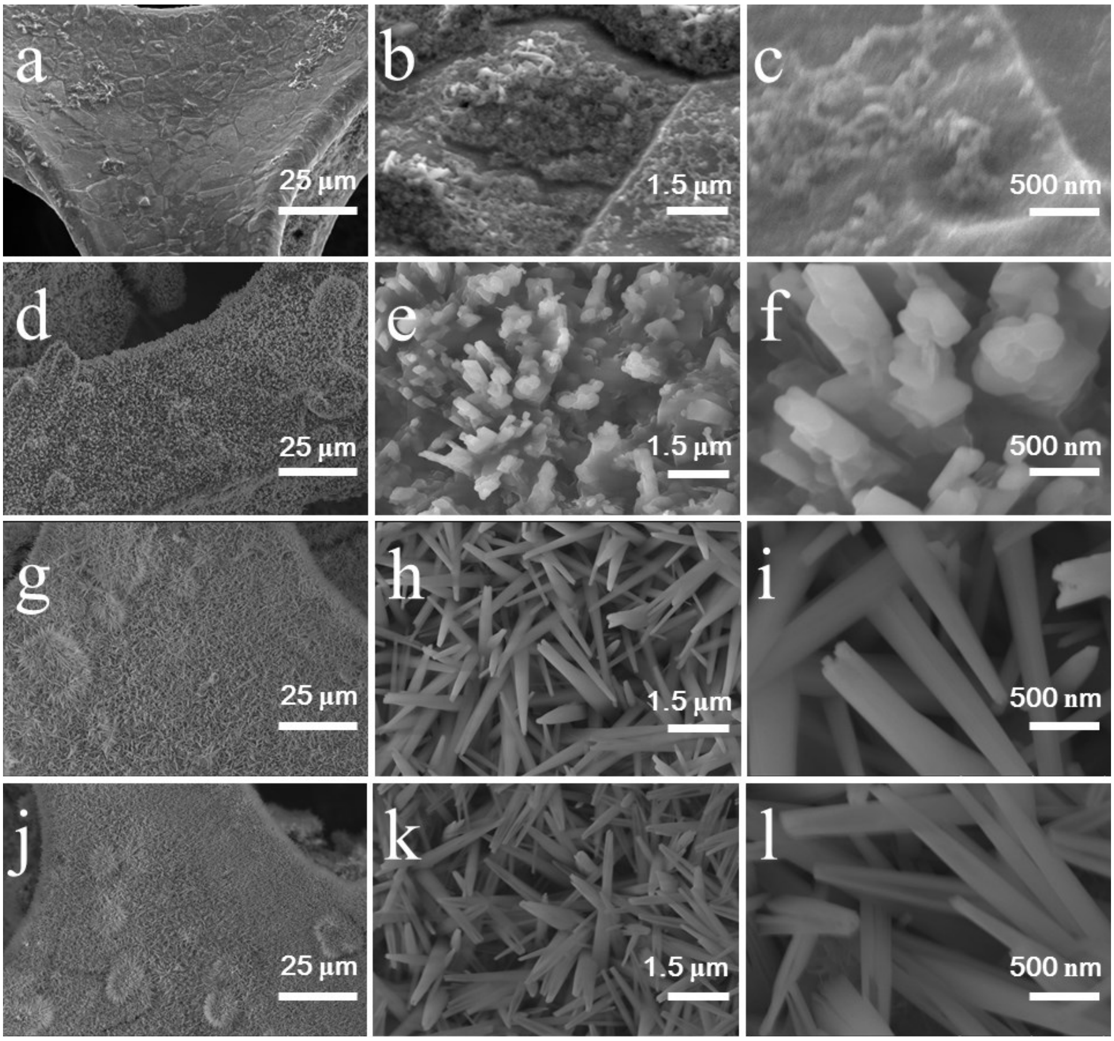

Figure S2. SEM image of CuO/rGO@NF electrodes prepared by electrochemical oxidation for 1 min (a-c) ,10 min (d-f), 30 min (g-i), 1 h (j-l)

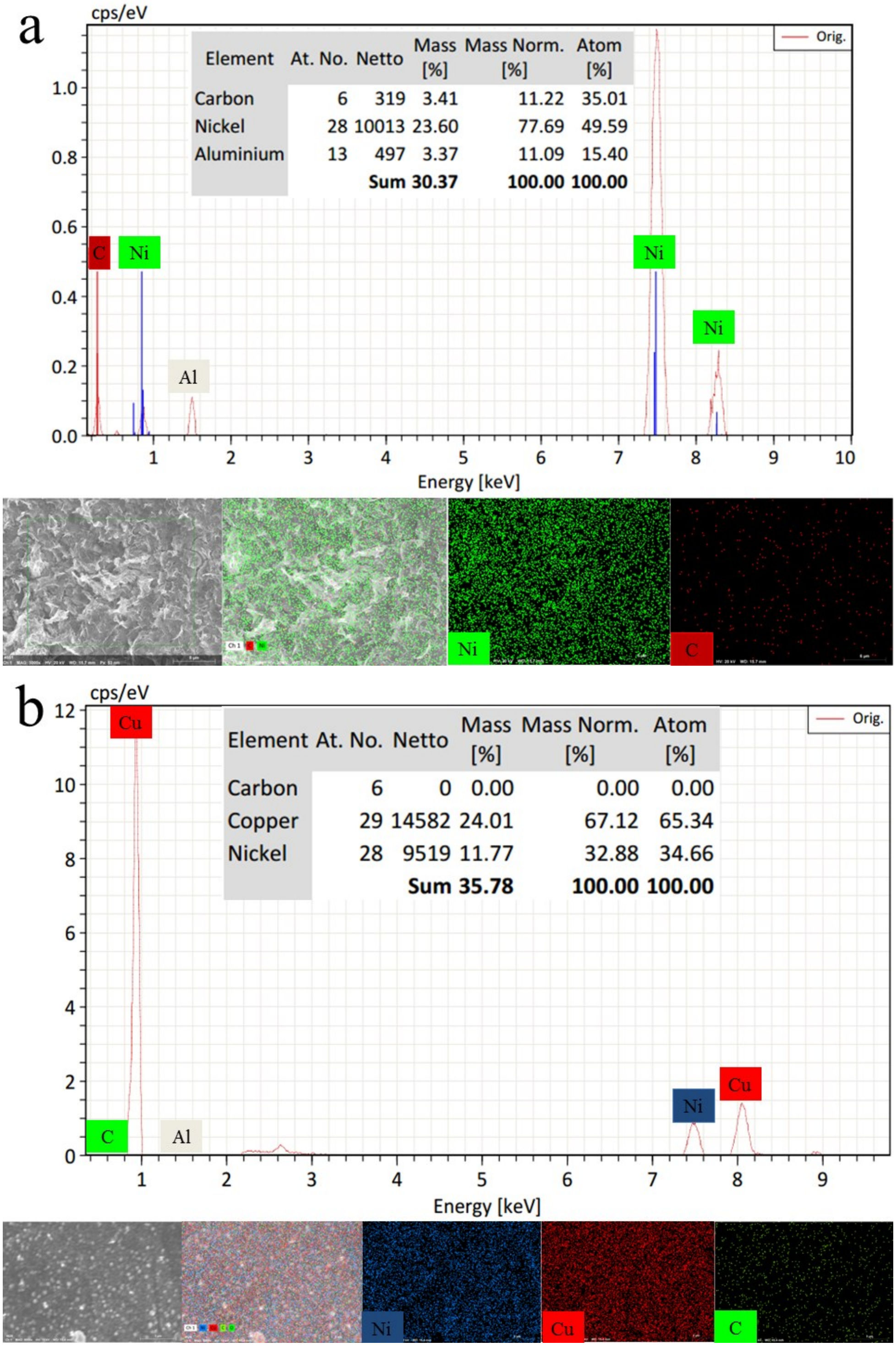

Figure S3. EDX image of (a) rGO@NF electrode and (b)Cu/rGO@NF electrode with different element mapping images

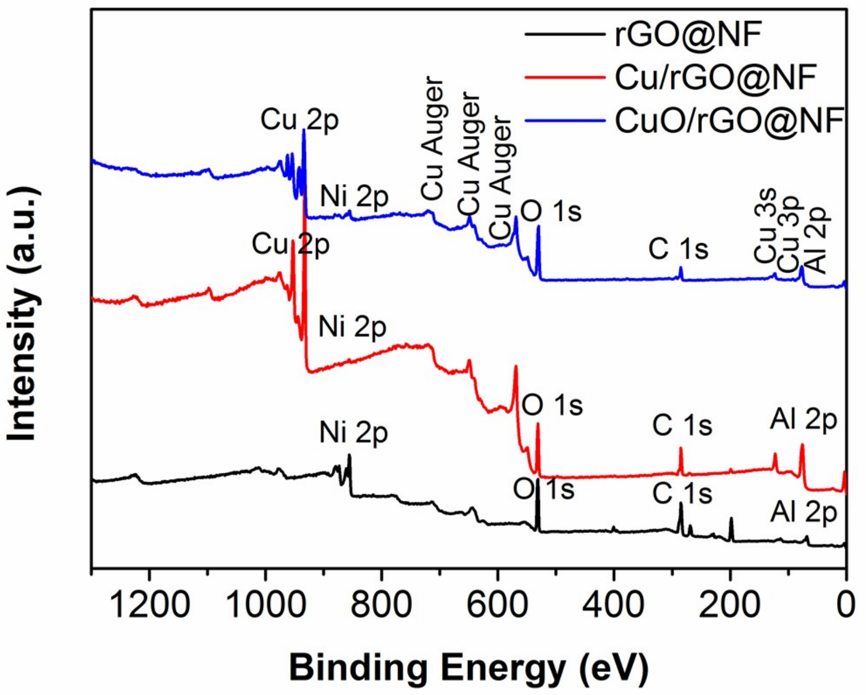

Figure S4. XPS survey spectrum of rGO@NF, Cu/rGO@NF and Cu/rGO@NF electrodes.

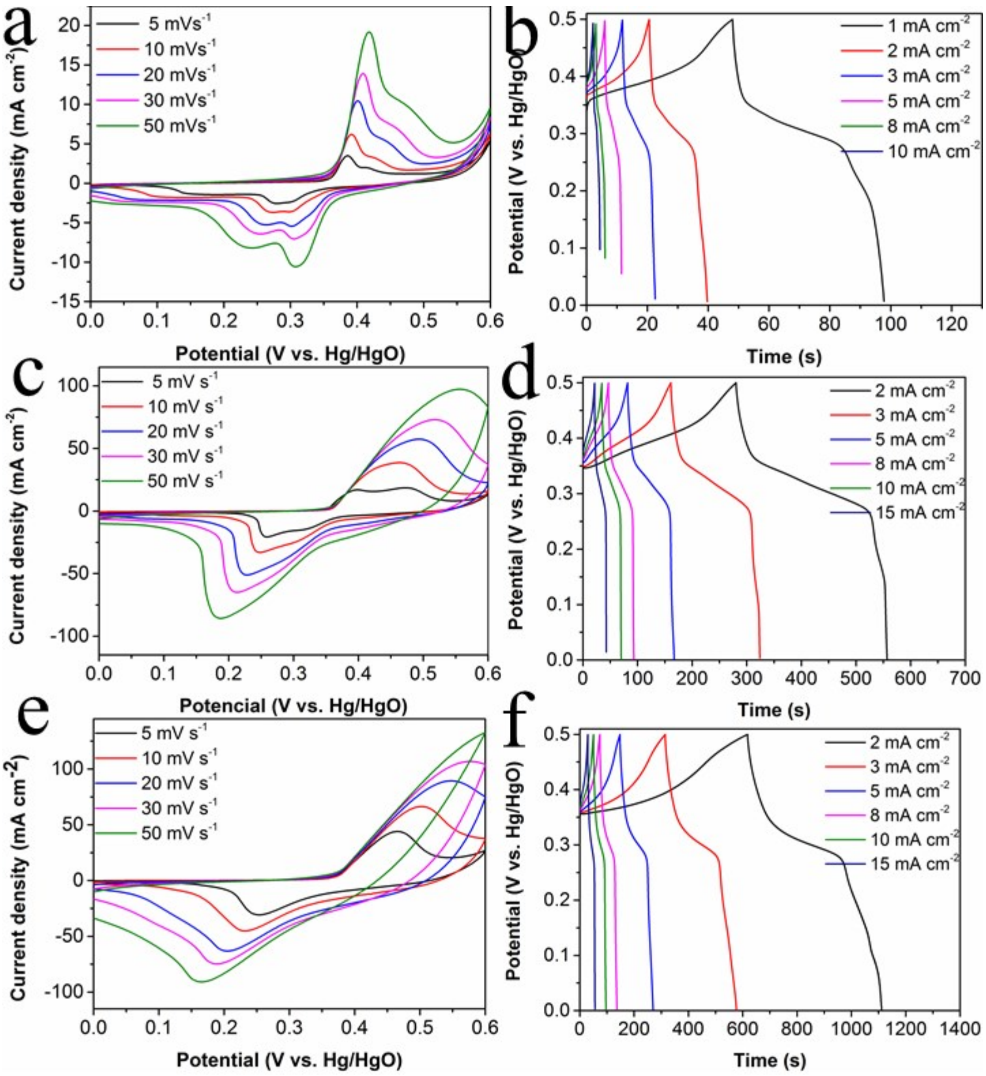

Figure S5. (a,c,e) CV curves of the NF, rGO@NF and Cu/rGO@NF electrodes at different scan rates. (b,d,f) GCD curves of the NF, rGO@NF and Cu/rGO@NF electrodes at various current densities.

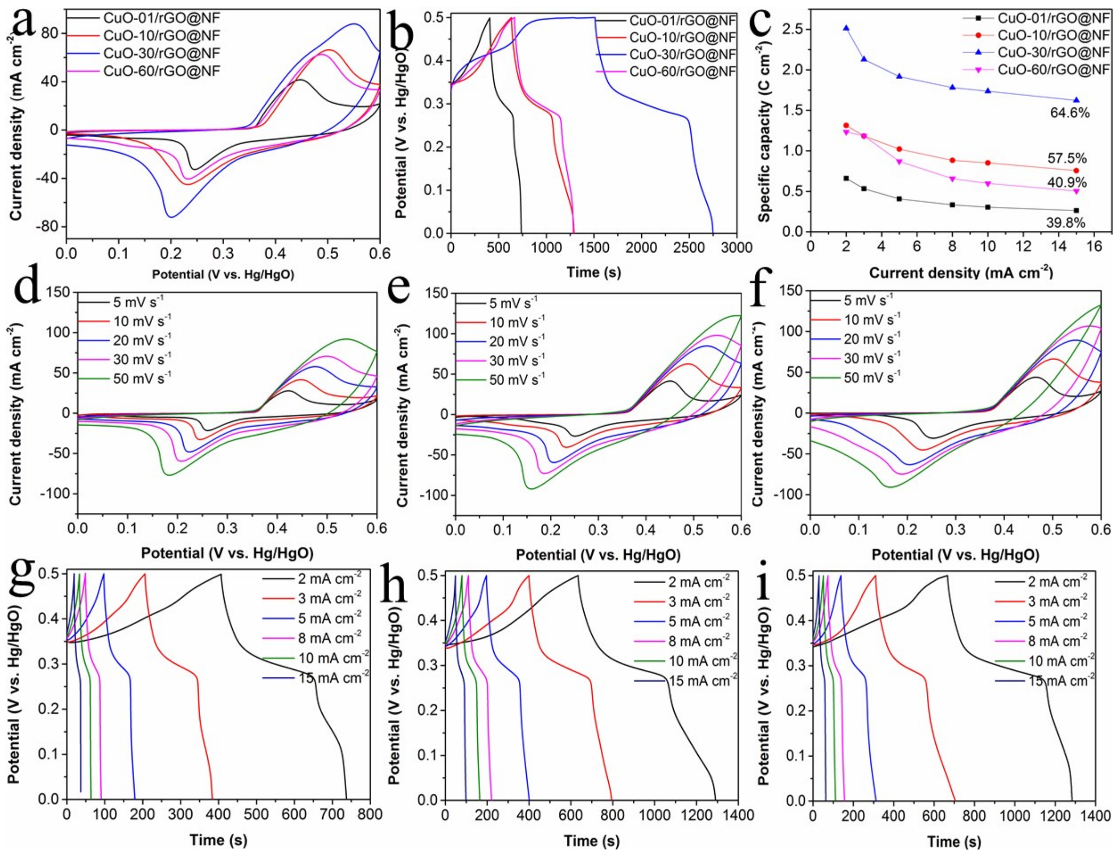

Figure S6. (a) Comparative CV curves of the CuO-01/rGO@NF, CuO-10/rGO@NF, CuO-30/rGO@NF, CuO-60/rGO@NF electrodes at 10 mV s^{-1}. (b) Comparative GCD curves of the four electrodes at 2 mA cm^{-2}. (c) Specific capacitance of the four electrodes at different current densities. (d-f) CV curves of the CuO-01/rGO@NF, CuO-10/rGO@NF, CuO-60/rGO@NF electrodes at different scan rates. (g-i) GCD curves of the CuO-01/rGO@NF, CuO-10/rGO@NF, CuO-60/rGO@NF electrodes at various current densities.

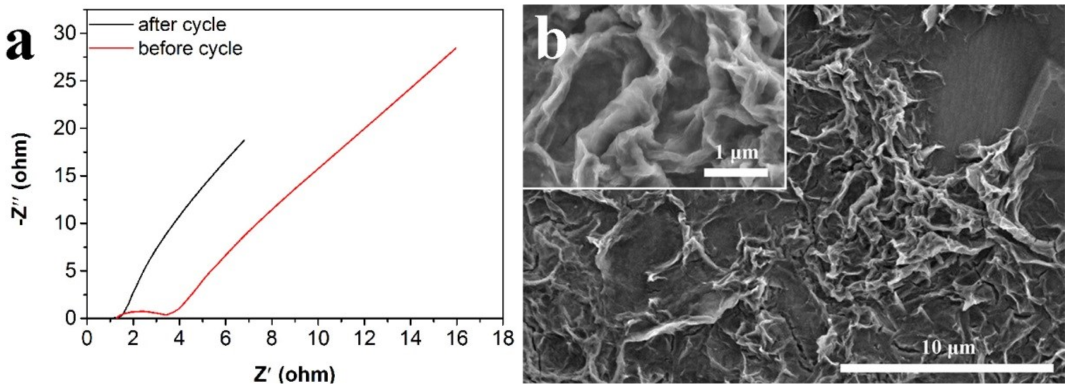

Figure S7. (a) EIS plots of the CuO/rGO@NF electrode before and after stability test at a frequency range of 100 kHz-0.01 Hz. (b) SEM image of CuO/rGO@NF after 1500 charge-discharge cycles.

Table S1. Comparison of the capacity of the similar materials reported previously.

Electrode	Method	Electrolyte	Capacity or Capacitance	Ref.
**MoNPs/NF@Ni_3S_2**	Ion implantation	2 M KOH	1.06 C cm^{-2} at 1 mA cm^{-2}	1
**Ni_3S_2/ NF**	Hydrothermal	2 M KOH	0.50 C cm^{-2} at 1 mA cm^{-2}	1
**NiO/Ni_3S_2**	Hydrothermal	6 M KOH	2.28 C cm^{-2} at 2 mA cm^{-2}	2
**$Cu(OH)_2$/Cu/CLS**	Copper Plating	6 M KOH	8.46 F cm^{-3} at 5 mA cm^{-3}	3
**Cu-MOF/$Cu_{2+1}O$**	Hydrothermal	6 M KOH	1.54 F cm^{-2} at 2 mA cm^{-2}	4
**3D CuO/Cu**	Femtosecond laser	3 M KOH	3.34 F cm^{-2} at 1 mA cm^{-2}	5
**CuO NSs-CWTs**	Drop-casting	1 M KOH	0.67 F cm^{-2} at 2 mA cm^{-2}	6
**CVO Cu@CuO**	CV oxidation	6 M KOH	1.67 F cm^{-2} at 2 mA cm^{-2}	7
**NiO-CuO**	Hydrothermal	3 M KOH	4.35 F cm^{-2} at 2 mA cm^{-2}	8
**Cu/Cu_2O**	Photo-assist	2 M KOH	782.0 F g^{-1} at 1 A cm^{-2}	9
**NiCo-LDH/CuO**	Corrosion growing	3 M KOH	1.97 F cm^{-2} at 7.96 A cm^{-2}	10
**CuO/rGO@NF**	Filtered cathodic vacuum arc technology	6 M KOH	2.51 C cm^{-2} at 2 mA cm^{-2}	This work

References

(1) Cheng, Y.; Zhai, M. M.; Guo, M. S.; Yu, Y. N.; Hu, J. B., A novel electrode for supercapacitors: Spicules-like Ni_3S_2 shell grown on molybdenum nanoparticles doped nickel foam. *Appl. Surf. Sci.* **2019,** *467*, 1113-1121.

(2) Yan, Z.; Guo, C.; Yang, F.; Zhang, C.; Mao, Y.; Cui, S.; Wei, Y.; Hou, L.; Xu, L., Cliff-like NiO/Ni_3S_2 Directly Grown on Ni Foam for Battery-type Electrode with High Area Capacity and

Long Cycle Stability. S0013468617317413.

(3) Chang, P.; Mei, H.; Zhao, Y.; Huang, W.; Zhou, S.; Cheng, L., 3D Structural Strengthening Urchin-Like $Cu(OH)_2$-Based Symmetric Supercapacitors with Adjustable Capacitance. **2019,** *29*, 1903588.

(4) Cao, X.; Cui, L.; Liu, B.; Liu, Y.; Jia, D.; Yang, W.; Razal, J. M.; Liu, J., Reverse synthesis of star anise-like cobalt doped Cu-MOF/$Cu_{2+1}O$ hybrid materials based on a Cu(OH)2 precursor for high performance supercapacitors. **2019,** *7*, 3815-3827.

(5) Wang, S.; Hu, J.; Jiang, L.; Li, X.; Cao, J.; Wang, Q.; Wang, A.; Li, X.; Qu, L.; Lu, Y., High–performance 3D CuO/Cu flowers supercapacitor electrodes by femtosecond laser enhanced electrochemical anodization. **2019,** *293*, 273-282.

(6) Cha, S. M.; Nagaraju, G.; Chandra Sekhar, S.; Yu, J. S., A facile drop-casting approach to nanostructured copper oxide-painted conductive woven textile as binder-free electrode for improved energy storage performance in redox-additive electrolyte. **2017,** *5*, 2224-2234.

(7) Liu, Y.; Cao, X.; Jiang, D.; Jia, D.; Liu, J., Hierarchical CuO nanorod arrays in situ generated on three-dimensional copper foam via cyclic voltammetry oxidation for high-performance supercapacitors. **2018,** *6*, 10474-10483.

(8) Fang, Z.; Rehman, S. u.; Sun, M.; Yuan, Y.; Jin, S.; Bi, H., Hybrid NiO–CuO mesoporous nanowire array with abundant oxygen vacancies and a hollow structure as a high-performance asymmetric supercapacitor. **2018,** *6*, 21131-21142.

(9) An, C. H.; Wang, Z. F.; Xi, W.; Wang, K.; Liu, X. Z.; Ding, Y., Nanoporous Cu@Cu_2O hybrid arrays enable photo-assisted supercapacitor with enhanced capacities. *J. Mater. Chem. A* **2019,** *7*, 15691-15697.

(10) Guo, Y.; Hong, X.; Wang, Y.; Li, Q.; Meng, J.; Dai, R.; Liu, X.; He, L.; Mai, L., Multicomponent Hierarchical Cu-Doped NiCo-LDH/CuO Double Arrays for Ultralong - Life Hybrid Fiber Supercapacitor. **2019,** *29*, 1809004.
